# The University of Michigan

**Published:** 1856-03

**Authors:** 


*The University of Michigan.*—We find in the February number of the Peninsular Journal of Medicine, the following paragraph:

"☞ At a sitting of the *Supreme Court* of the State of Michigan, in this city (Detroit) January 22, 1856, present a full bench,

"A motion was made by C. J. Walker, for the issuing of an alternative *mandamus*, compelling the Board of Regents of the University to appoint a Professor of Homœopathy according to the act of the legislature, creating the chair, or to show cause why the same is not done. The case is held under advisement, and also for further authorities in the case."

"☞ Since writing the above, we are informed that a *mandamus* has been granted."

We premise by saying that it cannot be doubted that the result of this action will be to force a chair of Homœopathy upon the University. We say it cannot be doubted, inasmuch as the conditions of the establishment of the school make it just and legal that such a chair should be established· The school is a State Institution. *All* of its people have a right to representation there, and those believing that homœopathy is the only true medical science, may demand that, in the school which belongs as much to them as to legitimate medicine, their doctrines should be taught.

It is with no feeling of exultation that we, for our own vindication, place on record this fulfilment of our prophecies made some years since, and recall to the memories of our readers the controversy to which they gave occasion. We should not deem it necessary to resort to this course, were it not that when that controversy closed we were driven from the field, not by argument, but by the most insulting personalities, accompanying charges of the most unmanly character. We waited for the event to sustain and vindicate our position, and that event has arrived.

To this matter of personalities we shall make only this allusion. The editorial management of both Journals has changed since then, and we should not inherit any quarrels save those of principle. It was in the month of October, 1853, that our first article on Medical Education appeared. We spoke of the voluntary competitional system as that best adapted to the country, and laid down as the characteristic points of that system:

"First—a complete independence from government control, or patronage.

"Second—an entire dependence on the respect and good-will of the profession for support.

"Third—an active competition of the schools with each other."

Further on, in arguing the possibility of the success of a government enterprize, we say:

"It is, then, proposed to adopt in this country, a system which has already proved a failure in Europe. Would it succeed better if transplanted to a republican soil? We have already sufficiently shown that the plan can only flourish in great capitals. In New York, in New Orleans, and possibly in Philadelphia, this condition could be complied with. But if family influence is so ruinous in Europe, would it be less so here? The government plan requires such permanency in the appointing power as can only be had in monarchical countries. The professorial appointments should be free from *political* changes, and their influence. In a word, as our politics are at present carried on by a system of partizan rewards and punishments, no state school will remain long free from this deteriorating influence; and with the constant changes in governors and cabinets, an equally constant change in teachers will obtain, and active political character be looked upon as the first requisite in a medical teacher. This is not a theoretical, but a practical and imminent danger; and the combination of family with political influence would result in the elevation of the sons of great men, and small practitioners, to the professorial chairs.

"The system of *concours* is one necessary to the well-being of government schools which will not be adopted in this country. We say will not, because it would clash with political intrigue, and party spoils. But were it adopted it is still subject to family and personal influence. It is the only, but the feeble guaranty against outside collusion. Without it such collusion is inevitable—with it, it is probable.

"A large salary is another condition of success. Talent has a cash value, and the salaries should be such as to fill the chairs with an array of superior men. A man of real eminence, of active energy, and true progress, will not for a thousand, or two thousand dollars yearly, consign himself to what must soon become the obscure and forgotten position of professor in a sleepy government school.

"Another thing may be mentioned: The opening of free schools of medicine would certainly have little tendency to improve the character of the profession. Medicine would become the refuge of broken down clergymen, and disgraced lawyers; of all those whose dislike to honest manual labor would lead them to avoid it. In a country like this, every high-spirited and honest young man should be able to enter the portals of the profession by the fruits of his own labor. The discipline of the struggle for success, and the manly feeling of pride which should reward that success, must be far preferable to the charity-boy feeling, inseparable from benefits unpaid for.

"We have thus enumerated the advantages attendant on our present system, and the disadvantages, viz: want of proper location and clinical teaching, the danger of political and family influence, the absence of the '*concours*' method of appointment, and of permanence and stability in the appointing power; the smallness of the salaries likely to be paid, and the consequent inferiority of the teaching; and finally, the loss of influence and character to the profession by the admission to it of unworthy members, attracted by the cheapness of the diploma. Until these objections are overcome, and this requirement complied with, no state school should hold a position of equality with those worth paying for—better taught, and better supplied with the collateral means of instruction."

It is now between two and three years since we advanced these sentiments. We allude to it, inasmuch as we were pretty lonely in our position then. We expected a lively controversy with the spirited "Peninsular," but we did *not* expect that a half a dozen of our exchanges, whom we might enumerate, would one by one utter a growl, at our arguments, and impeach our motives. "Small classes at Buffalo" was considered a phrase of infinite meaning just then. Since that time all of these aforesaid exchanges have reversed their position. They have been driven to fight our battles for us, and when we have seen them pretty soundly cudgelled for inconsistency by the "Peninsular," we can't say that we have been among the mourners.

But we may say now a word in vindication of the University of Buffalo, and our action as its volunteer representative. The Buffalo school, more than any other, is the offspring of individual enterprize. Two thousand dollars covers the whole of its receipts from the State, and yet it has better buildings, better museums, better clinics, and—saving ourselves—as good instructors as any other. It has ever rested, not upon its cheapness, but, its merits. Placed in a city where the expenses of living are heavy, exacting larger fees than any other school at the East outside of New York and Philadelphia, placed near enough these schools to be in immediate competition with them for the support of students seeking the highest advantages, and having on its other side a gratuitous school to compete with it for the poorer class, it has still, we are proud to say, maintained as high a standard of graduation as any other in the land, and never sacrificed the interests of the profession to its own prosperity.

Under these circumstances it has maintained its average attendance better than many other schools, and the attendance of the past winter—larger than that of the preceding — teaches us that we have as yet no reason for discouragement, but every reason to hope for a brilliant future.

